# Simultaneous characterization of sense and antisense genomic processes by the double-stranded hidden Markov model

**DOI:** 10.1093/nar/gkv1184

**Published:** 2015-11-17

**Authors:** Julia Glas, Sebastian Dümcke, Benedikt Zacher, Don Poron, Julien Gagneur, Achim Tresch

**Affiliations:** 1 Gene Center Munich and Department of Biochemistry, Ludwig-Maximilians-Universität München, Feodor-Lynen-Straße 25, 81377 Munich, Germany; 2 Department of Plant Breeding and Genetics, Max Planck Institute for Plant Breeding Research, Carl-von-Linné-Weg 10, 50829 Cologne, Germany; 3 Institute for Genetics, University of Cologne, Zülpicher Str. 47b, 50674 Cologne, Germany

## Abstract

Hidden Markov models (HMMs) have been extensively used to dissect the genome into functionally distinct regions using data such as RNA expression or DNA binding measurements. It is a challenge to disentangle processes occurring on complementary strands of the same genomic region. We present the double-stranded HMM (dsHMM), a model for the strand-specific analysis of genomic processes. We applied dsHMM to yeast using strand specific transcription data, nucleosome data, and protein binding data for a set of 11 factors associated with the regulation of transcription.The resulting annotation recovers the mRNA transcription cycle (initiation, elongation, termination) while correctly predicting strand-specificity and directionality of the transcription process. We find that pre-initiation complex formation is an essentially undirected process, giving rise to a large number of bidirectional promoters and to pervasive antisense transcription. Notably, 12% of all transcriptionally active positions showed simultaneous activity on both strands. Furthermore, dsHMM reveals that antisense transcription is specifically suppressed by Nrd1, a yeast termination factor.

## INTRODUCTION

The rapidly growing amount of heterogeneous data generated by experimental high-throughput techniques makes integrative data analysis an essential part of molecular biology. One major purpose is the creation of comprehensive views on high dimensional data which are impossible to obtain manually. To that end, clustering methods group data points into a finite number of distinct ‘groups’ or ‘states’, according to some measure of similarity. Our present work considers the analysis of data from several experiments whose output can be aligned to a genomic sequence, such as RNA expression-, ChIP- or DNA methylation data. The main purpose is to cluster genomic positions into ‘states’, which ideally correspond to distinct biological functions. Hidden Markov models (HMMs) have become the method of choice, since they additionally account for the dependency of consecutive observations introduced by the sequential structure of the data. HMMs were successfully used for dissecting the genome into ‘chromatin states’ (1) or ‘transcription states’ (2). Recently, HMMs were employed to infer distinct genomic states from genome-wide ChIP data in human (1,3–8), fly (9,10), Arabidopsis (11) and worm (12,13). However, the drawback of the HMMs used in these applications is their inability to integrate stand-specific (e.g. RNA expression) with non-strand-specific (e.g. ChIP) data and thus limiting the analysis either to only non-strand-specific or only single-stranded data. This limitation was first addressed in (14). There, the hidden Markov chain is replaced by a dynamic Bayesian network (DBN). This allows the modeling of strand-specific data and the introduction of structured states, i.e. hierarchical labels for each position. Other approaches employed reversible HMMs (15), which were further extended by the bidirectional hidden Markov model (2). Still all of these models are unable to account for overlapping processes that might occur on both DNA strands. This situation is frequently encountered in compact genomes. For example, cryptic unstable transcripts (CUTs) and stable uncharacterized transcripts (SUTs) often overlap with annotated features (16). Neil *et al*. (17) showed that by far the most CUTs in yeast are antisense CUTs, i.e. CUTs that are transcribed from between tandem features in antisense direction.

In the present work we introduce double-stranded hidden Markov models (dsHMMs), which explicitly model the forward and reverse DNA strand by two Markov chains running in opposite directions. Therefore dsHMMs are able to disentangle the two strands at every single position of the genome. dsHMMs capture the biology of directed genomic processes such as transcription, which often occur at the same position on both strands. We illustrate the use of dsHMMs on a data set comprised of strand-specific expression, nucleosome occupancy and ChIP-chip data of 11 factors involved in yeast transcription. We present the first strand-specific map of transcription states in yeast. Our results confirm the role of Nrd1 in a non-canonical pathway of transcription termination, which predominately occurs in antisense direction of stable gene transcripts and is mainly involved in the termination of CUTs (16,18).

## MATERIALS AND METHODS

### Definition of the dsHMM

**Figure 1.**
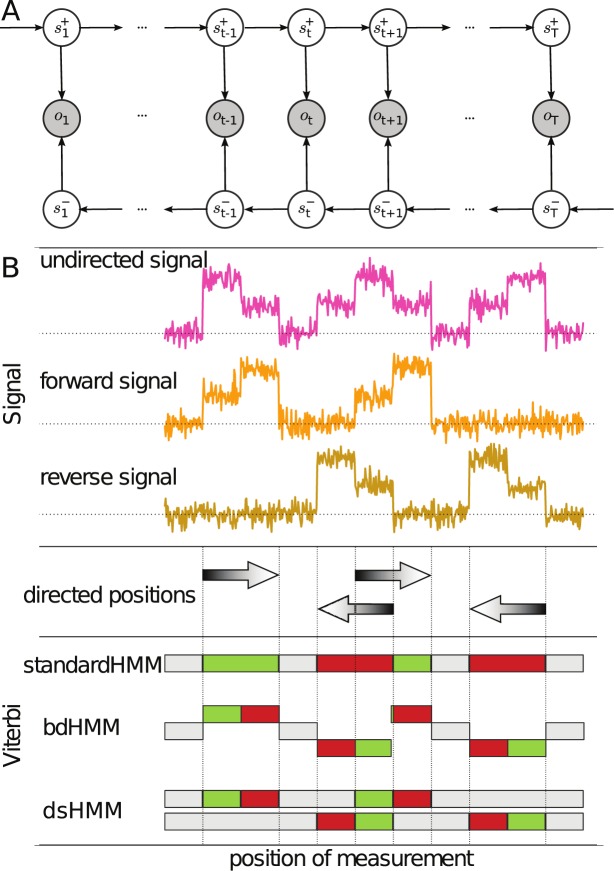
(**A**) Graphical representation of a dsHMM showing two hidden state chains $\lbrace s_{1}^{+},\dots ,s_{T}^{+}\rbrace$ and $\lbrace s_{1}^{-},\dots ,s_{T}^{-}\rbrace$ which run in opposite direction (white circles). Each state pair $(s_{t}^{+},s_{t}^{-})$ emits an observation *o*_*t*_, *t* = 1, ..., *T* (gray circles). (**B**) Viterbi paths inferred from a synthetic data set using three different HMM models. The top panel shows a simulated ChIP experiment (purple track) and an RNA-Seq experiment with forward (orange track) and reverse strand (brown track) expression data for a genomic region containing partly overlapping genes (arrows in the middle panel) located on both DNA strands. The bottom panel shows the Viterbi paths obtained from the standard HMM, the bdHMM and the dsHMM with three hidden states: an intergenic state (gray) and two gene-specific states (red and green). For the bdHMM, equivalent forward and reverse states are indicated by the same color, positioned either above (forward direction) or below (reverse direction) the undirected gray states.

Let *P* be a set of genome-wide experiments (‘tracks’) which give rise to a sequence of observables $\mathcal {O}=(o_{1},...,o_{T})$, $o_{t}\in \mathbb {R}^{P}$, where $o_{t}^{j}$ contains the measurement value of track *j* at position *t*. The observables are emitted by two independent, homogeneous Markov chains of hidden variables running in opposite direction, the forward chain $\mathcal {S}^{+}=(s_{1}^{+},...,s_{T}^{+})$ and the reverse chain $\mathcal {S}^{-}=(s_{1}^{-},...,s_{T}^{-})$. The idea is that among a finite set of biological processes $\mathbb {D}$, each state $s_{t}^{+}\in \mathbb {D}$ (respectively $s_{t}^{-}\in \mathbb {D})$ indicates which process is taking place on the forward (respectively reverse) DNA strand. The emission distribution of an observation *o*_*t*_ is conditionally independent of all other observations, given the hidden state pair $(s_{t}^{+},s_{t}^{-})$. A graphical specification of the double-stranded hidden Markov model (dsHMM) is given in Figure 1A. According to our assumptions, the joint likelihood of a dsHMM factors into

(1) \begin{equation*} P(\mathcal {O},\mathcal {S}^{+},\mathcal {S}^{-})=P(\mathcal {O}\mid \mathcal {S}^{+},\mathcal {S}^{-})\cdot P(\mathcal {S}^{+})\cdot P(\mathcal {S}^{-}) \end{equation*}

(2) \begin{equation*} P(\mathcal {O}\mid \mathcal {S}^{+},\mathcal {S}^{-})=\prod _{t=1}^{T}P(o_{t}\mid s_{t}^{+},s_{t}^{-}) \end{equation*}

(3) \begin{equation*} \begin{split} P(\mathcal {S}^{+})=P(s_{1}^{+})\prod _{t=2}^{T}P(s_{t}^{+}\mid s_{t-1}^{+}), \\ P(\mathcal {S}^{-})=P(s_{T}^{-})\prod _{t=2}^{T}P(s_{t-1}^{-}\mid s_{t}^{-}) \end{split} \end{equation*}

It is natural to assume that state transitions happening in forward direction on the forward strand have the same probability as state transitions on the reverse strand happening in reverse direction, i.e.

(4) \begin{equation*} P(s_{t}^{+}=j\mid s_{t-1}^{+}=i)=P(s_{t-1}^{-}=j\mid s_{t}^{-}=i)=a_{ij}\quad ,\ i,j\in \mathbb {D} \end{equation*}

for some transition matrix $A=(a_{ij})\in \mathbb {R}^{\mathbb {D}\times \mathbb {D}}$. By the same reasoning, we assume

(5) \begin{equation*} P(s_{1}^{+}=i)=P(s_{T}^{-}=i)=\pi _{i}\quad ,\ i\in \mathbb {D} \end{equation*}

for some initial state distribution $\pi \in \mathbb {R}^{\mathbb {D}}$. For convenience, we require that *A* is ergodic, and that π is its unique steady state distribution. A dsHMM can then be transformed into a standard HMM with state space $\mathbb {D}^{2}=\mathbb {D}\times \mathbb {D}$, with state sequence $\mathcal {S}=(s_{1},...,s_{T})$, $s_{t}=(s_{t}^{+},s_{t}^{-})\in \mathbb {D}^{2}$. The transition matrix $B=(b_{rs})\in \mathbb {R}^{\mathbb {D}^{2}\times \mathbb {D}^{2}}$ becomes

(6) \begin{equation*} b_{rs}=a_{r^{+}s^{+}}a_{s^{-}r^{-}}\pi _{s^{-}}\pi _{r^{-}}^{-1}\quad ,\ r=(r^{+},r^{-}),\,s=(s^{+},s^{-})\in \mathbb {D}^{2} \end{equation*}

and the initial state distribution, $\tau \in \mathbb {R}^{\mathbb {D}^{2}}$ is $\tau _{s}=\pi _{s^{+}}\pi _{s^{-}}$ (see Supplementary Data Part I Section 1 for details). The dsHMM is a reversible HMM (see Supplementary Data Part I Section 1 Remark 3). The transformation of the dsHMM into a standard HMM will allow us to apply well-known, efficient techniques for HMM learning, namely the Forward-Backward, Viterbi and Baum–Welch algorithms (see Supplementary Data Part I Sections 3–5).

It remains to find a sparse parametrization of the emission distributions ψ_*s*_(*o*) = *P*(*o*∣*s*), $s\in \mathbb {D}^{2}$. Let the observation space $\mathbb {R}^{P}$ be the Cartesian product of the strand-unspecific observations $\mathbb {R}^{B}$, and the forward- respectively reverse strand-specific observations $\mathbb {R}^{E^{+}}$ and $\mathbb {R}^{E^{-}}$, i.e. let *P* = *B*∪*E*^+^∪*E*^−^, where *E*^+^ and *E*^−^ are disjoint copies from a set *E*. We assume that $\psi _{s}(o)\sim \mathcal {N}(o;\nu ^{s},\Gamma ^{s})$ is a multivariate Gaussian with mean $\nu ^{s}=(\nu _{p}^{s})$ and covariance matrix Γ^*s*^ which is built from ‘strand-specific’, multivariate Gaussian emission distributions $\varphi _{i}(o)\sim \mathcal {N}(o;\mu ^{i},\Sigma ^{i})$, $i\in \mathbb {D}$, with mean $\mu ^{i}\in \mathbb {R}^{B\cup E}$ and covariance matrix Σ^*i*^. Let $P_{s}^{+}=\left\lbrace b\in B\mid \mu _{b}^{s^{+}}\ge \mu _{b}^{s^{-}}\right\rbrace \cup E^{+}$the set of tracks in which the forward strand process *s*^+^ ‘dominates’ over *s*^−^, and vice versa, let $P_{s}^{-}=\left\lbrace b\in B\mid \mu _{b}^{s^{+}}<\mu _{b}^{s^{-}}\right\rbrace \cup E^{-}$. We define ν^*s*^ by

(7) \begin{equation*} \nu _{p}^{s}=\left\lbrace \begin{array}{@{}l@{\quad }l@{}}\mu _{p}^{s^{+}} & \mbox{if }p\in P_{s}^{+}\\ \mu _{p}^{s^{-}} & \mbox{if }p\in P_{s}^{-} \end{array}\right.\quad ,\ p\in P,\,s\in \mathbb {D}^{2} \end{equation*}

The most natural choice for Γ^*s*^, $s=(s^{+},s^{-})\in \mathbb {D}^{2}$, in the sense that the largest possible parts of the marginal covariance structures from $\Sigma ^{s^{+}}$ and $\Sigma ^{s^{-}}$ are maintained, is

(8) \begin{equation*} \Gamma _{p_{1}p_{2}}^{(s)}=\left\lbrace \begin{array}{@{}l@{\quad }l@{}}\Sigma _{p_{1}p_{2}}^{(s^{+})} & \textrm {if}\,p_{1},p_{2}\in P_{s}^{+}\\ \Sigma _{p_{1}p_{2}}^{(s^{-})} & \textrm {if}\,p_{1},p_{2}\in P_{s}^{-}\\ 0 & \textrm {else} \end{array}\right.\quad ,\ p_{1},p_{2}\in P,\,s\in \mathbb {D}^{2} \end{equation*}

(see Supplementary Data Part I Section 2 for details). Be aware that the choice of the mean vectors ν^*s*^ (Equation 7) is a critical step in the dsHMM construction process, since it determines how fictitious forward and reverse strand-specific measurements $\mu _{p}^{s^{+}}$, $\mu _{p}^{s^{-}}$ are combined to one value $\nu _{p}^{s}$. Equation 7 also leads to a non-trivial, yet exact and efficient update formula for the means in the EM algorithm, which contributes to the usability of the dsHMM. Other definitions led to inferior results, for reasons given in the Supplementary Data Part I Section 6. As a consequence of this sparse parametrization, our dsHMM has exactly the same number of parameters as a standard HMM with state space $\mathbb {D}$ and Gaussian emission distributions on a single-stranded observation space $\mathbb {R}^{B\cup E}$. For the initialization of the EM algorithm, the means μ^*j*^ of the transcription states $j\in \mathbb {D}$ were determined by k-means clustering. First, genomic positions are classified according to their transcriptional activity into a set of transcriptionally active and inactive regions, respectively. The set of regions showing transcription on the forward strand is used to cluster a defined number of transcribed states and the set of transcriptionally inactive regions is used to cluster a defined number of intergenic states. The covariance matrices Σ^*j*^ are set to the empirical covariance matrix of the observations that were clustered to the respective transcription state $j\in \mathbb {D}$ and not updated throughout the EM algorithm. The initial state distribution π and the transition matrix *A* are initialized uniformly (see Supplementary Data Part I for details).

### Experimental Data

#### ChIP–chip data

The data used in this work comprise ChIP–chip data sets of various proteins and strand-specific expression data. The selected proteins cover most of the factors involved in the mRNA transcription cycle. They include initiation factors, different types of elongation factors as well as termination factors. Additionally, tracks of Rpb3, a subunit of Pol II, and some of its phosphorylated forms, typical for certain phases of transcription were used. The data were taken from Mayer *et al*. (19,20). The data include measurements for Rpb3, a PolII subunit, several CTD modifications (S5P, S7P, Y1P and S2P), TFIIB, a general initiation factor, the elongation factors Spt5, Bur1 and Spn1 and the termination factors Nrd1 and Pcf11 (see Supplementary Data Part II Table S2). Furthermore, we included ChIP–chip measurements of the nucleosome occupancy from Lee *et al*. (21).

Expression data were taken from Xu *et al*. (16). They used tiling arrays to profile the transcriptome in yeast. In addition to the profiling of wild-type variants in various media, they profiled the transcriptome for a deletion mutant of *RRP6*. Profiling of the deletion mutant enabled the detection of CUTs since *RRP6* is necessary for their usually rapid degradation. CUTs as well as SUTs often overlap with annotated features. We decided to use the data set of the deletion mutant as we designed the model explicitly to deal with overlapping transcription. Consequently, expression data of the mutant offer a good opportunity to check the quality of the model.

#### Data preprocessing

Since ChIP–chip experiments show high levels of systematic noise, normalization of the data is essential. A general problem is caused by the execution of protocols. Due to the complex execution of ChIP–chip experiments, it is inevitable that small changes occur between the different samples. As even subtle changes have an influence on the results, it is necessary to analyze and correct for differences between the single data sets (22).

Further, noise is caused by the chemical properties of DNA. A base pair consisting of adenine and thymine has two hydrogen bonds, while a base pair consisting of cytosine and guanine has three hydrogen bonds. This results in different binding affinities dependent on the base composition. One possibility to correct for this bias is the subtraction of a Mock IP or genomic input DNA, as these reflect the binding affinities of the pure DNA (22).

The ChiP–chip data we used in this work were normalized according to the protocol suggested by Zacher *et al*. (23). This involved applying the *Starr* package (22) published in the Bioconductor project (24). In order to make them comparable, data sets were finally rescaled. The 5% quantile was set to 0, and the 95% quantile was set to 1.

## RESULTS

### A model for strand-specific genome annotation

Compact genomes often utilize both complementary strands of the DNA to simultaneously perform distinct tasks. Even in larger genomes, enhancer sites of genes located on the forward strand may be located in intronic regions of genes transcribed in reverse direction, creating regions with alternate functions of the forward and reverse strand. This overlap of functions poses a challenge to genome annotation algorithms. To this end, we have developed the double-stranded hidden Markov model (dsHMM) for strand-specific genome annotation (Figure 1A). Figure 1B illustrates the situation at the example of a synthetic data set. We model four genes, two in forward and two in backward direction, including an overlap between two genes. The data consist of one undirected signal (representing e.g. protein–DNA binding), and two directed signals (representing e.g. transcriptional activity). The undirected signal is consistent within active regions in forward and reverse direction. It starts with a high signal and ends up with a low signal. We trained a standard HMM, a bdHMM (2) and our new dsHMM on these data assuming three hidden states, one intergenic state and two gene states. The resulting three Viterbi annotations are shown in the lower panel of Figure 1B. The standard HMM is not able to use the strand-specific information and learns one active state in forward direction, one active state in reverse direction and one inactive state. It cannot distinguish between the beginning and the end of active regions. At the overlapping region it decides for one of the active states as it cannot model the overlap appropriately. Increasing the number of states in the standard HMM would solve the problem of distinguishing between the start and the end of active regions, but lead to another problem. Without an explicit coupling the model would assign completely independent states to regions of forward and reverse direction, without exploiting the similarity between forward and reverse processes. This inflates the number of parameters in the model and does not use strand-specifity of information in a meaningful way.

In contrast to the standard HMM the bdHMM correctly recognizes directionality and distinguishes between the beginning and the end of active regions (Figure 1B). The bdHMM detects the equivalence between regions of forward and reverse directionality by assigning corresponding forward and reverse states to them. But the overlap of genes cannot be appropriately modeled as the bdHMM merely assigns one state to each position, and this state is either a forward, reverse or undirected state. On our synthetic data, the bdHMM first annotates a reverse state within the overlapping region and then switches to the corresponding forward state, ignoring the transcriptional activity on the complementary strand.

The dsHMM model assumes that the observed sequence of states is emitted by two Markov chains, a forward and a backward chain that run in opposite direction (Figure 1A, see Materials and Methods and Supplementary Data Part I for a formal definition of dsHMMs). The dsHMM resolves these issues by assigning a pair of states to each position. This pair represents the biological processes on both strands. In our example (Figure 1B), the model annotates the correct sequence of states for both strands. It distinguishes between the start and the end of active regions and has learned directed transition probabilities. In the overlapping region active states are assigned to both the forward and the reverse strand.

### The dsHMM dissects the RNA transcription cycle in yeast

**Figure 2.**
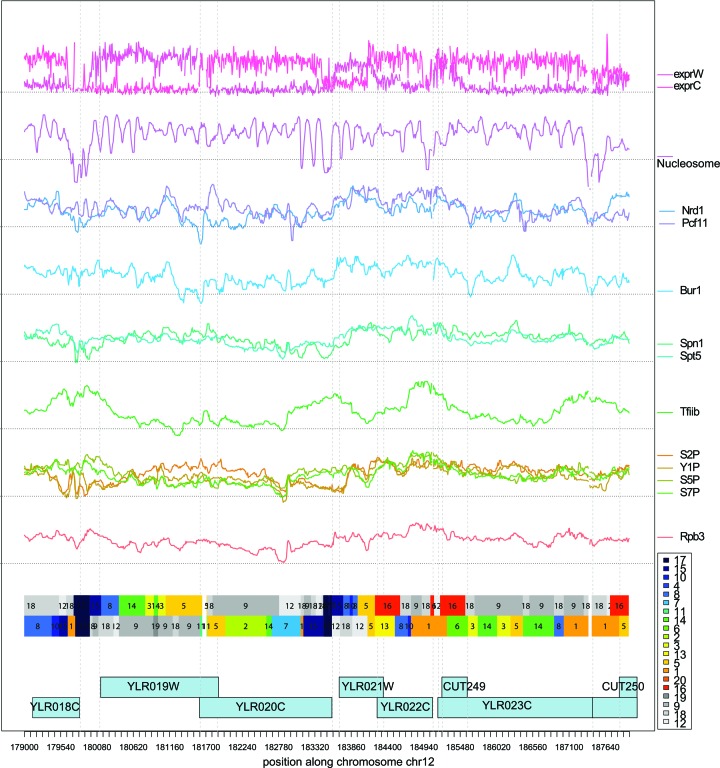
Genomic region on yeast chromosome 12 and Viterbi annotation of the dsHMM. At the top all input data are shown (forward and reverse transcription profiles from RNA-Seq experiments, nucleosome occupancies, ChIP–chip profiles of factors involved in transcription and termination: Nrd1, Pcf11, Bur1, Spn1, Spt5, TFIIB and the CTD phosporylations S2P, Y1P, S5P, S7P and last the PolII subunit Rbp3. The Viterbi paths of the dsHMM is shown with the color codes of the hidden states in the right margin. At the bottom we show the genes and CUTs annotated to this genomic region. The x-axis shows the position along chromosome 12.

**Figure 3.**
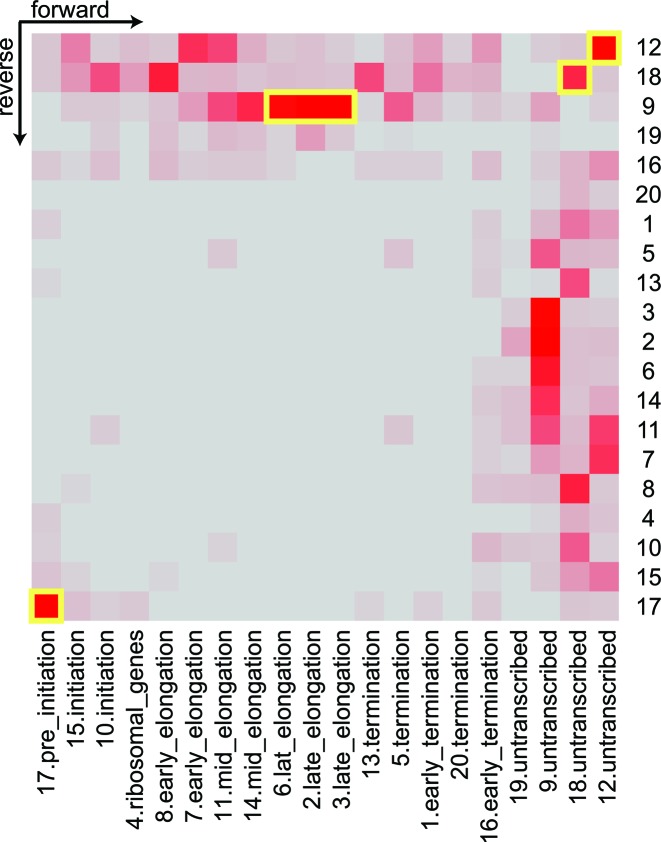
The symmetric heatmap of the frequency of state pair (*x, y*) respectively (*y, x*) annotated to the (forward,reverse) strand for the 20 states of the dsHMM. Regions without transcription are annotated by state 12,18 on both strands (yellow highlight, top right corner). Interestingly state 17, the pre-initiation state is annotated simultaneously on both strands, indicating that a region of transcription initiation generally gives rise to transcription on both strands (yellow highlight, bottom left corner). While the transcription is in elongation phase (states 6, 2, 3), state 9 (no transcription) is predominantly annotated to the opposite strand (yellow highlight).

**Figure 4.**
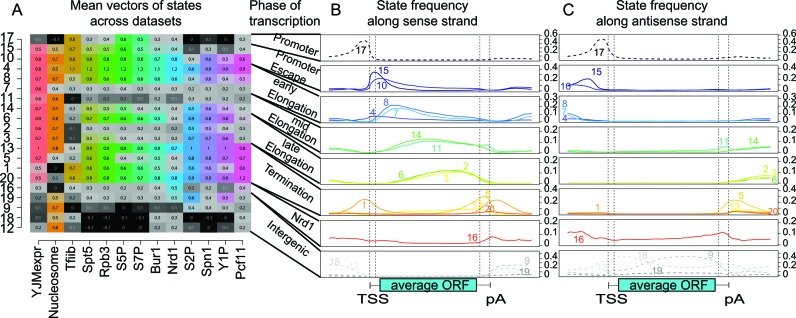
Transcription states of the dsHMM learned on the yeast data. The left panel shows the mean values emitted by all 20 states (rows) for each measurement data track (columns). For each of the 20 states, their spatial state distribution along the sense strand (middle panel) and antisense strand (right panel) of an average transcript was calculated from the Viterbi paths of 4362 representative genes. Directed states are in color, undirected states in gray. Dashed lines are used for states with a low expression mean value <0.2.

**Figure 5.**
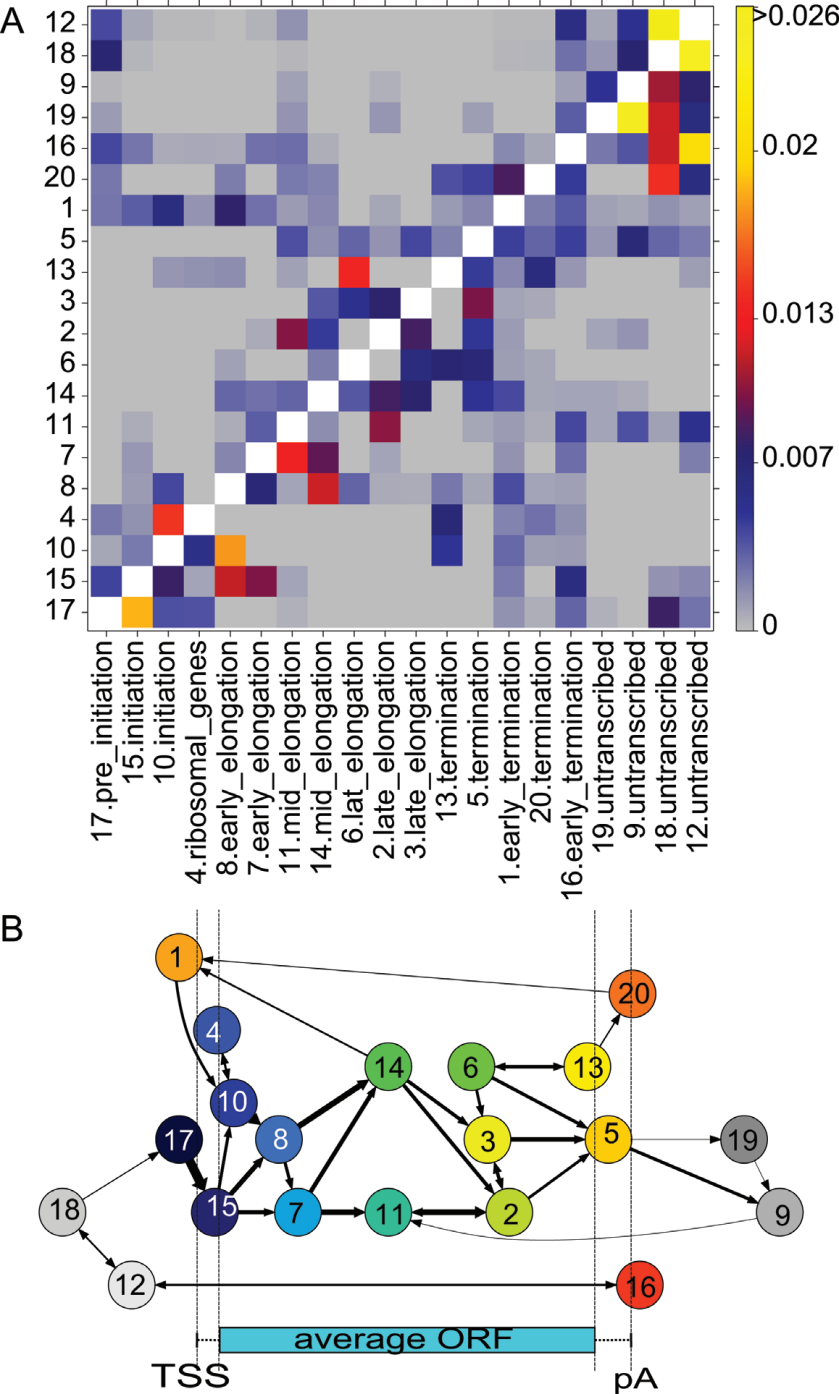
(**A**) Transition matrix of the dsHMM trained on expression and ChIP–chip data of yeast. Diagonal elements are consistently above 0.9 and were blanked out. (**B**) Visualization of state transitions on the sense strand of genes. States are positioned according to the point where their state frequency peaks. The thickness of an arrow *i* → *j* corresponds to the relative frequency of the observed state transitions *i* → *j* on the Viterbi path on the sense strand of genes. For clarity, arrows corresponding to a transition probability lower than 0.012 are omitted.

We learned a dsHMM with 20 states (i.e. 400 state pairs) on strand specific expression data, nucleosome occupancy data and on ChIP–chip data of 11 factors involved in yeast transcription (see Materials and Methods and Supplementary Data Part II Table S2 for a description of the data). As already stated by other authors (7), we emphasize that the number of states in the model is largely a matter of choice. Statistical model selection criteria like the Akaike information criterion or the Bayesian information criterion do not apply, because they were designed to yield an optimal fit to the data in terms of (predictive) precision. Given a large data set as in the present case, they suggest an exceedingly high number of states (6). However, since the dsHMM is used as an exploratory method, the results need to be interpretable. Thus, the number of states should be small enough to be inspected manually, and the emission distributions of different states should be sufficiently different to have a distinct biological interpretation. The main output of the dsHMM learning process is an annotation of the genome (Figure 2) with hidden state pairs (Figure 3), together with the parameters of the dsHMM, namely the state-specific emission distributions and the state transition probabilities. In the annotation of the genome we observe that states such as states 17, 15, 8, 14 occur in an ordered fashion along known transcripts, whereas states 19, 9, 18 and 12 (in gray in Figure 2) are typically annotated on the opposite strand. The inspection of the emission mean vectors (Figure 4A) reveals that there are 15 states (colored) corresponding to transcribed regions with polymerase activity, and 5 transcriptionally silent states (gray). Mapping the transcribed states to their peak position on the metagene, together with their most frequent transitions defines the canonical transcription cycle (colored blue through green to red in Figure 5) as defined by (19). Visualizing the frequency at each position along an idealized transcript helps the interpretation of the HMM states (see Figure 4B,C and Supplementary Data Part II Table 1). We compared our dsHMM annotation to an annotation learned by the bdHMM model on the same data, using the same number of (strand-specific) states and the same initialization (Supplementary Data Part III Figure 2). Expectedly, the agreement of bdHMM states and sense strand dsHMM states was very good, whereas it was poor between bdHMM states and antisense dsHMM states (see the discussion below Supplementary Data Part III Figure 2).

**Table 1.** Contingency table of the states representing untranscribed regions (states 12, 18, 9, 19) and those annotating a transcriptional signal. Only 6.9% of the yeast genome does not have any transcribed elements. 80.9% are annotated to be transcriptionally active on at least on strand. Interestingly 12.2% of the genome is annotated as transcriptionally active simultaneously on both strand

		Reverse strand	
		Untranscribed	Transcribed
Forward strand	Untranscribed	6.9%	40.8%
	Transcribed	40.1%	12.2%

### Transcription initiation has low directional preference

It recently turned out that the direction of transcription initiation is much less biased as previously thought (16,17,25). In yeast this results in a large number of bidirectional promoters, leading to genes transcribed in head-to-head conformation (called divergent transcripts). Xu *et al*. (16) showed that 48% of protein-coding transcripts and 61% of unannotated transcripts with a nucleosome depleted 5′ region are initiated from a bidirectional promoter. The Viterbi annotation of the yeast genome by the dsHMM identifies three states as typical transcription initiation states (state 17, 10 and 15), characterized by low nucleosome occupancy and high mean occupancy of TFIIB and by their occurrence centered around the transcription start site (Figure 4B,C first two panels). State 17 peaks at the identical position in the Viterbi annotation of both forward and reverse strand. There are 5336 regions in the yeast genome annotated by State 17 (2648 and 2688 and the Waston and Crick strands, respectively), with 2124 regions overlapping on both strands. This corresponds to 4248 genomic features transcribed from regions annotated simultaneously to be in State 17, the pre-initation state, suggesting that the signals for pre-initiation complex formation are not strand specific. States 10 and 15 peak roughly 50 bp downstream of state 17 on the forward strand, and roughly 50 bp upstream of state 17 on the reverse strand. States 10 and 15 are characterized by the presence of Polymerase 2 conjointly with its S5P and S7P CTD early elongation phosphorylation marks ((19) and Suplementary Data Part II Table 1). Remarkably the early elongation peaks in antisense direction of the annotated transcript are almost as pronounced as the canonical forward peaks, confirming the pervasiveness of bidirectional transcription. To investigate this phenomenon in an unbiased way, we calculated the contingency table of forward and reverse state annotation for the states 12, 18, 9 and 19 representing untranscribed regions and the remaining states (Table 1). This shows that most of the yeast genome is transcribed (93.1% of genomic position are annotated by a transcribed state on at least on strand) with 12.2% of genomic positions being annotated as transcriptionally active on both strands. A total of 6.9% of the genome seems to be transcriptionally silent.

### Antisense transcription is suppressed by an alternative termination pathway

There are three states peaking around the termination site (states 1, 5 and 16, disregarding the infrequent states 13 and 20). State 5 is interpreted as a termination state (see Supplementary Data Part II Table 1) and it is the only termination state annotated to regions of overlapping transcriptional activity (in 24.1% of the cases when state 5 was annotated, a transcriptionally active state was assigned to the opposite strand). It peaks at the pA site of genes and in the corresponding 3′ region on the antisense strand (see Figure 4). Figure 2 shows two examples of genes in tail-to-tail conformation (gene pairs YLR019W/YLR020C and YLR021W/YLR022C) and the corresponding dsHMM annotation. State 1 shows mixed occupancy of initiation and termination factor occupancies and is therefore also detected at the TSS of the metagene. This typically identifies genes that follow each other in quick succession on the genome (tandem conformation).

Of all single state combinations that model overlapping transcriptional activity, combinations including state 16 are by far the most frequent. Most notably, it is the only state whose frequency on the antisense strand is generally higher than on the sense strand. On the antisense strand, state frequency of state 16 shows two peaks, one upstream of the promoter and one around the pA site Figure 4C. Both peaks can be explained by antisense transcription, which is known to preferentially originate at regions of transcription initiation and termination (16). The mean occupancy profile of state 16 shows low but existing levels of expression, consistent with antisense transcripts. Moreover, the distinguishing feature of state 16 is its high Nrd1 occupancy. This is perfectly in line with the fact the Nrd1 is responsible for the termination of CUTs (17). The model was able to separate the Nrd1 signal from the signals arising from the sense strand. This is a clear improvement over previous models since standard HMMs do not have the possibility to consider the processes of forward and reverse strand separately.

## DISCUSSION

In this paper we developed a model, called dsHMM, for the unbiased annotation of the genome. Its distinctive features are its ability to handle strand-specific data, recognize directionality and model overlapping transcriptional activity. We trained a dsHMM on the basis of 12 ChIP–chip tracks (undirected signals) and transcriptional activity data (directed signal). These data comprised transcription factors necessary for all phases of transcription, various modifications of both Pol II and nucleosomes. It was shown that the hidden states of the learned model accurately recovered the different phases of the transcription cycle. Each phase is characterized by typical protein binding patterns. Consequently, the trained dsHMM has successfully captured the important biological characteristics of the transcription process. The separate modeling of the two DNA strands finds bidirectional promoters and distinguishes between the canonical and the Nrd1 assisted transcription termination pathways. Most remarkably the Nrd1 pathway is most frequently annotated in antisense direction to coding genes and peaks shortly before TSS and after the pA site of these coding genes. This suggest the role of Nrd1 as the main factor for the suppression of antisense transctiption (18).

Formally the dsHMM operates on hidden state pairs which grow quadratically in the number of strand specific states. By introducing natural symmetry constraints, we ensure that the number of parameters is the same as in a standard HMM. While this is uncritical for the transition matrix (see Equation 6), we need to make additional assumptions for the emission distributions (see Equation 7 and Supplementary Data Part I Section 2). The emission distribution of nucleosomes get a special treatment. To capture also the biologically important depletion of nucleosomes at an early state of transcription we define the transcribed state as the one determining nucleosome occupancy in tuple states consisting of a transcribed and an intergenic state. Otherwise a higher nucleosome occupancy of an intergenic state would mask the depletion of the transcribed state. In all remaining tuple combinations, i.e. transcribed versus transcribed and intergenic versus intergenic, we used Equation 7.

We envisage that the creation of a joint emission distribution can be improved for count data which is on an absolute scale (e.g. sequencing data) as opposed to the relative scale of microarray measurements used in this study. Further we did not learn or update the covariance structure for each state but rather conservatively fixed the covariance structure of the emission distribution to the empirical covariance structure of the whole data.

So far, we have only included data of some transcription factors, various modifications of Pol II and nucleosomes into the model. It is tempting to include data of different activating and repressing histone modifications. This will help to elucidate the interplay between transcription factors and chromatin marks. A promising application is the annotation of plant genomes, as plant Pol4 and Pol5 establish a transcriptional feedback to chromatin structure (26). dsHMM could also shed light on the epigenomic/epigenetic silencing mechanisms of retrotransposons that frequently lie on the antisense strand of intronic regions of genes (27).

## SUPPLEMENTARY DATA

Supplementary Data are available at NAR online.

SUPPLEMENTARY DATA
