# Supplementary material for: Simultaneous characterization of sense and antisense genomic processes by the double-stranded hidden Markov model
Source: Nucleic Acids Res. 2015 Nov 17;44(5):e44. doi: 10.1093/nar/gkv1184 (PMC4797261; doi:10.1093/nar/gkv1184)
Supplement: SUPPLEMENTARY DATA [file supp_44_5_e44__index.html]

Simultaneous characterization of sense and antisense genomic processes by the double-stranded hidden Markov model — SUPPLEMENTARY DATA 

# Simultaneous characterization of sense and antisense genomic processes by the double-stranded hidden Markov model

## SUPPLEMENTARY DATA

- SUPPLEMENTARY DATA
