# Supplementary material for: Simultaneous characterization of sense and antisense genomic processes by the double-stranded hidden Markov model
Source: Nucleic Acids Res. 2015 Nov 17;44(5):e44. doi: 10.1093/nar/gkv1184 (PMC4797261; doi:10.1093/nar/gkv1184)
Supplement: SUPPLEMENTARY DATA [file supp_gkv1184_nar-01713-met-n-2015-File010.pdf]

Simultaneous characterization of sense and antisense genomic processes by the double-stranded hidden Markov model

by Julia Glas, Sebastian Dümcke, Benedikt Zacher, Don Poron, Julien Gagneur, and Achim Tresch

October 16, 2015

Part I

Supplemental Methods

This supplement contains the detailed methods for the learning of the dsHMM. The dsHMM model and the learning algorithms are implemented in C++ and integrated in a general R/Bioconductor [8] framework for handling HMMs ([12]).

1 Conversion of a dsHMM into a standard HMM

In order to define the necessary algorithms for HMM inference and learning (forward-backward algorithm, Viterbi algorithm and Baum-Welch algorithm) we convert the dsHMM with two Markov chains into a standard HMM with a single Markov chain. Recall the definition of a dsHMM from the main text: Given a sequence of observations $\mathcal{O} = (o_1, ..., o_T)$, $o_t \in \mathbb{R}^P$, and two sequences of hidden states, $\mathcal{S}^+ = (s_1^+, ..., s_T^+)$ and $\mathcal{S}^- = (s_1^-, ..., s_T^-)$, $s_t^+, s_t^- \in \mathbb{D}$, the likelihood function is

$$P(\mathcal{O}, \mathcal{S}^+, \mathcal{S}^-) = P(\mathcal{O} \mid \mathcal{S}^+, \mathcal{S}^-) \cdot P(\mathcal{S}^+) \cdot P(\mathcal{S}^-) \tag{1}$$

The individual factors in Equation (1) decompose further into

$$P(\mathcal{O} \mid \mathcal{S}^+, \mathcal{S}^-) = \prod_{t=1}^{T} P(o_t \mid s_t^+, s_t^-) = \prod_{t=1}^{T} \psi_{(s_t^+, s_t^-)}(o_t) \tag{2}$$

with emission distributions $\psi_{(s^+, s^-)}(o)$, and

$$\begin{aligned} P(\mathcal{S}^+) &= P(s_1^+) \prod_{t=2}^{T} P(s_t^+ \mid s_{t-1}^+) = \pi_{s_1^+} \prod_{t=2}^{T} a_{s_{t-1}^+ s_t^+} \\ P(\mathcal{S}^-) &= P(s_T^-) \prod_{t=2}^{T} P(s_{t-1}^- \mid s_t^-) = \pi_{s_T^-} \prod_{t=2}^{T} a_{s_t^- s_{t-1}^-} \end{aligned} \tag{3}$$

for some state transition matrix $A = (a_{ij}) \in \mathbb{R}^{\mathbb{D} \times \mathbb{D}}$ and some initial state distribution $\pi \in \mathbb{R}^{\mathbb{D}}$. We require that A is ergodic, and that π is its unique steady state distribution. Let $\mathcal{S} = (s_1, ..., s_T)$, $s_t = (s_t^+, s_t^-) \in \mathbb{D}^2$. Due to Markov

equivalence in Bayesian networks [10], the reverse Markov chain also factorizes in forward order:

$$\begin{aligned} P(\mathcal{S}^-) &= P(s_T^-) \cdot \prod_{t=2}^{T} P(s_t^- \mid s_{t-1}^-) \\ &= P(s_T^-) \cdot \prod_{t=2}^{T} \left(P(s_t^- \mid s_{t-1}^-) \cdot \frac{P(s_{t-1}^-)}{P(s_t^-)} \right) \\ &= P(s_1^-) \prod_{t=2}^{T} P(s_t^- \mid s_{t-1}^-) \end{aligned} \tag{4}$$

It follows that $\mathcal{S}$ is a Markov chain, since

$$\begin{aligned} P(\mathcal{S}) &= P(\mathcal{S}^+) \cdot P(\mathcal{S}^-) \\ &= P(s_1^+) \cdot P(s_1^-) \cdot \prod_{t=2}^{T} \left(P(s_t^+ \mid s_{t-1}^+) P(s_t^- \mid s_{t-1}^-) \right) \\ &= P(s_1) \cdot \prod_{t=2}^{T} P(s_t \mid s_{t-1}) \end{aligned} \tag{5}$$

The transition matrix $B = (b_{rs})_{r,s \in \mathbb{D}^2}$ for this Markov chain is

$$\begin{aligned} b_{rs} &= P(s_t = s \mid s_{t-1} = r) = P(s_t^+ = s^+ \mid s_{t-1}^+ = r^+) \cdot P(s_t^- = s^- \mid s_{t-1}^- = r^-) \\ &= a_{r^+s^+} \cdot P(s_{t-1}^- = r^- \mid s_t^- = s^-) \frac{P(s_t^- = s^-)}{P(s_{t-1}^- = r^-)} \\ &= a_{r^+s^+} a_{s^-r^-} \pi_{s^-} \pi_{r^-}^{-1} \end{aligned} \tag{6}$$

In (6), we exploited that π is the stationary state of A, and therefore $P(s_t^- = s^-) = \pi_{s^-} > 0$ for all $t = 1, ..., T$ and all $s^- \in \mathbb{D}$. The initial state distribution $\tau = (\tau_s)_{s \in \mathbb{D}^2}$ is

$$\tau_s = P(s_1 = s) = P(s_1^+ = s^+) P(s_1^- = s^-) = \pi_{s^+} \pi_{s^-} \tag{7}$$

The likelihood function (1) has therefore the form of a standard HMM with state space $\mathbb{D}^2$, emission distributions $\Psi = \{\psi_s; s \in \mathbb{D}^2\}$, initial state distribution τ and transition matrix B,

$$\begin{aligned} P(\mathcal{O}, \mathcal{S}^+, \mathcal{S}^-) &= P(\mathcal{O} \mid \mathcal{S}^+, \mathcal{S}^-) \cdot P(\mathcal{S}^+) \cdot P(\mathcal{S}^-) \\ &= \prod_{t=1}^{T} \psi_{s_t}(o_t) \cdot \tau_{s_1} \cdot \prod_{t=2}^{T} b_{s_{t-1}s_t} \end{aligned} \tag{8}$$

A graphical representation of the standard HMM representation of a dsHMM is given in Figure 1.

Remarks

Remark 1. B is ergodic: Since A is ergodic, there is a positive integer K such that for all $k \geq K$, there exists to every two states $j_\alpha, j_\omega \in \mathbb{D}$ a path (= a sequence of states in $\mathbb{D}$) $j_1 = j_\alpha, j_2, ..., j_k = j_\omega$ such that $a_{j_{t-1}j_t} > 0$, $t = 2, ..., k$. Given two states $s_\alpha = (s_\alpha^+, s_\alpha^-)$, $s_\omega = (s_\omega^+, s_\omega^-) \in \mathbb{D}^2$, and some $k \geq K$, there exist paths $j_1^+ = s_\alpha^+, ..., j_k = s_\omega^+$ and $j_1^- = s_\omega^-, ..., j_k = s_\alpha^-$ such that $a_{j_{t-1}^+ j_t^+} > 0$, $a_{j_{t-1}^- j_t^-} > 0$, $t = 2, ..., k$. Define $s_t = (j_t^+, j_{k-t+1}^-)$, $t = 1, ..., k$. It follows that $s_1 = s_\alpha$, $s_k = s_\omega$, and $b_{s_{t-1}s_t} = a_{j_{t-1}^+ j_t^+} a_{j_{t-1}^- j_t^-} \pi_{j_{t-1}^-} \pi_{j_t^-}^{-1} > 0$, $t = 2, ..., k$, since all entries of π are positive by ergodicity of A. We have proved the ergodicity of B.

Remark 2. The distribution τ is the stationary state of B, since for $s \in \mathbb{D}^2$,

$$\begin{aligned} (\tau B)_s &= \sum_{r \in \mathbb{D}^2} \tau_r b_{rs} = \sum_{r \in \mathbb{D}^2} \pi_{r^+} \pi_{r^-} \cdot a_{r^+s^+} a_{s^-r^-} \pi_{s^-} \pi_{r^-}^{-1} \\ &= \sum_{r^+ \in \mathbb{D}} \pi_{r^+} \cdot a_{r^+s^+} \sum_{r^- \in \mathbb{D}} a_{s^-r^-} \pi_{s^-} \\ &= \pi_{s^-} \pi_{s^-} = \tau_s \end{aligned} \tag{9}$$

Remark 3. The Markov chain $\mathcal{S}$ is reversible (i.e., for all $r, s \in \mathbb{D}^2$, $\tau_r b_{rs} = \tau_s b_{sr}$ holds), and hence the dsHMM is a reversible HMM:

$$\begin{aligned}\tau_r b_{rs} &= \pi_{r^-}\pi_{r^+} \cdot a_{r^+s^+}a_{s^-r^-}\pi_{s^-}\pi_{r^-}^{-1} \\ &= \pi_{r^+} \cdot \left(a_{s^+r^+}\frac{\pi_{s^+}}{\pi_{r^+}}\right) \cdot \left(a_{r^-s^-}\frac{\pi_{r^-}}{\pi_{s^-}}\right) \cdot \pi_{s^-} \\ &= \pi_{s^+}\pi_{s^-} \cdot a_{s^+r^+}a_{r^-s^-}\pi_{r^-}\pi_{s^-}^{-1} \\ &= \tau_s b_{sr} \end{aligned} \tag{10}$$

Reversibility is a natural symmetry condition in the analysis of genomics data. It is a consequence of the plausible biological assumption that the same biological processes take place in forward and reverse direction with (approximately) the same frequency. Hence, state transitions from r to s should be observed at the same frequency as state transitions from s to r, as formalized in Equation (10).

2 Sparse parametrization of the dsHMM

By Equations (6) and (7), $B \in \mathbb{R}^{\mathbb{D}^2 \times \mathbb{D}^2}$ and $\tau \in \mathbb{R}^{\mathbb{D}^2}$ have a sparse parametrization derived from $A \in \mathbb{R}^{\mathbb{D}\times\mathbb{D}}$ (and $\pi \in \mathbb{R}^{\mathbb{D}}$, which, by assumption, is determined as the unique stationary state of A). It remains to find a sparse parametrization of the emission distributions $\psi_s(o) = P(o \mid s)$, $s \in \mathbb{D}^2$. Let the observation space $\mathbb{R}^P$ be the Cartesian product of the strand-unspecific observations $\mathbb{R}^B$, and the forward- respectively reverse strand-specific observations $\mathbb{R}^{E^+}$ and $\mathbb{R}^{E^-}$, i.e., let $P = B \cup E^+ \cup E^-$, where E^+ and E^- are disjoint copies from a set E. We assume that $\psi_s(o) \sim \mathcal{N}(o; \nu^s, \Gamma^s)$ is a multivariate Gaussian with mean $\nu^s = (\nu_p^s)$ and covariance matrix Γ^s which are built from 'strand-unaware', multivariate Gaussian emission distributions $\Phi = \{\varphi_i;\, i \in \mathbb{D}\}$, $\varphi_i(o) \sim \mathcal{N}(o; \mu^i, \Sigma^i)$, with mean $\mu^i \in \mathbb{R}^{B\cup E}$ and covariance matrix Σ^i. Let $P_s^+ = \left\{b \in B \mid \mu_b^{s^+} \geq \mu_b^{s^-}\right\} \cup E^+$ the set of tracks in which the forward strand process s^+ 'dominates' over s^-, and vice versa, let $P_s^- = \left\{b \in B \mid \mu_b^{s^+} < \mu_b^{s^-}\right\} \cup E^-$. We define ν^s by

$$\nu_p^s = \begin{cases} \max\{\mu_p^{s^+}, \mu_p^{s^-}\} & \text{if } p \in B \\ \mu_p^{s^+} & \text{if } p \in E^+ \\ \mu_p^{s^-} & \text{if } p \in E^- \end{cases} = \begin{cases} \mu_p^{s^+} & \text{if } p \in P_s^+ \\ \mu_p^{s^-} & \text{if } p \in P_s^- \end{cases}, \; p \in P,\, s \in \mathbb{D}^2 \tag{11}$$

To illustrate and motivate this definition, let us consider two measurement tracks, a ChIP track and an RNA expression track. Suppose that in state $s^+ \in \mathbb{D}$, we predict a mean ChIP occupancy of 1, and an RNA expression of 2, $\mu^{s^+} = (1,2)^T$. For a second state $s^- \in \mathbb{D}$, we predict $\mu^{s^-} = (0,3)^T$. If we knew that at some genomic position, the biological process corresponding to s^+ (respectively s^-) is taking place on the forward (respectively reverse) strand, and we could separately measure forward and reverse RNA expression in two different tracks, we would predict, according to (11), a mean value $\nu^s = \nu^{(s^+,s^-)} = (1,2,3)^T$. Here, the second (third) entry of the vector ν^s represents the forward (reverse) RNA expression. The advantages and disadvantages of this choice will become more clear after we have established the EM algorithm for parameter learning. We will discuss them in Supplemental Part I Section 6, together with alternative definitions for ν^s.

The most natural choice for Γ^s, $s = (s^+, s^-) \in \mathbb{D}^2$, in the sense that the largest possible parts of the marginal covariance structures from Σ^{s^+} and Σ^{s^-} are maintained, is

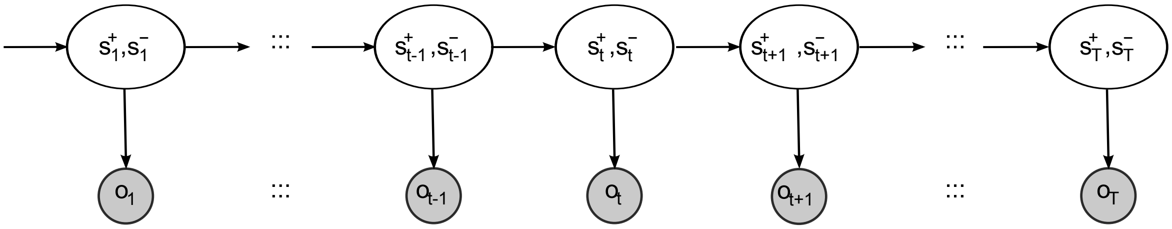

Figure 1: The dsHMM transformed into a standard HMM.

$$\Gamma^{(s)}_{p_1p_2} = \begin{cases} \Sigma^{(s^+)}_{p_1p_2} & \text{if } p_1, p_2 \in P^+_s \\ \Sigma^{(s^-)}_{p_1p_2} & \text{if } p_1, p_2 \in P^-_s \\ 0 & \text{else} \end{cases} \quad , \; p_1, p_2 \in P, \; s \in \mathbb{D}^2 \tag{12}$$

With these choices, a dsHMM is parametrized by $\theta = (A, \Phi)$ (again, we omit the mention of the redundant parameter π). The number of parameters equals that of a standard HMM with strand-unaware observations $o_t \in \mathbb{R}^{B \cup E}$ and strand-unspecific state space $\mathbb{D}$ (assuming that the initial state distribution is given by the stationary state of the ergodic matrix A).

3 The forward-backward algorithm for dsHMMs

We briefly recall the notation and the algorithms from standard HMM theory as they can for instance be found in [9] or [1]. Let $\theta = (A, \Phi)$ be a parametrization of a dsHMM, and let B, τ, Ψ be derived from θ according to (6), (7), (11), and (12). The forward-backward algorithm efficiently marginalizes the joint probability distribution of an HMM over the distribution of the hidden state variables. Since we have transformed the dsHMM into a standard HMM, the recursive formulas for the calculation of the forward and backward probabilities apply: For all $s \in \mathbb{D}^2$,

$$\begin{aligned} \alpha_t(s) &:= P(o_1, ..., o_t, s_t = s \mid \theta) = \sum_{r \in \mathbb{D}^2} \alpha_{t-1}(r) \cdot b_{rs} \cdot \psi_s(o_t) \\ \alpha_1(s) &:= P(o_1, s_1 = s \mid \theta) = \tau_j \cdot \psi_j(o_1) \end{aligned} \tag{13}$$

$$\begin{aligned} \beta_t(s) &:= P(o_t, ..., o_T, s_t = s \mid \theta) = \sum_{r \in \mathbb{D}^2} \beta_{t+1}(r) \cdot a_{sr} \cdot \psi_r(o_t) \\ \beta_T(s) &:= 1 \end{aligned} \tag{14}$$

The marginal likelihood $P(\mathcal{O} \mid \theta)$ can be calculated as

$$P(\mathcal{O} \mid \theta) = \sum_{s \in \mathbb{D}^2} P(o_1, ..., o_T, s_T = s \mid \theta) = \sum_{s \in \mathbb{D}^2} \alpha_T(s) \tag{15}$$

We need to define the terms $\gamma_t(s)$ and $\xi_t(r, s)$, $r, s \in \mathbb{D}^2$, which will be used in the next section.

$$\gamma_t(s) := P(s_t = s \mid \mathcal{O}, \theta) = \frac{\alpha_t(s) \cdot \beta_t(s)}{\sum_{r \in \mathbb{D}^2} \alpha_t(r) \cdot \beta_t(r)} \quad , \; t = 1, ..., T \tag{16}$$

$$\xi_t(r, s) := P(s_t = r, s_{t+1} = s \mid \mathcal{O}, \theta) = \frac{\alpha_t(r) \cdot a_{rs} \cdot b_s(o_{t+1}) \cdot \beta_{t+1}(s)}{\sum_{u \in \mathbb{D}^2} \alpha_t(u) \cdot \beta_t(u)}, \quad , \; t = 1, ..., T-1 \tag{17}$$

4 The Baum-Welch algorithm for dsHMMs

We derive a learning algorithm for dsHMMs which uses the maximum likelihood principle for the estimation of the model parameters. The aim is to locally maximize the marginal likelihood $P(\mathcal{O} \mid \theta)$. In standard HMM theory, this is accomplished by the Baum-Welch algorithm, an Expectation Maximization (EM) algorithm that has been tailored to HMMs. It consists of an E-step, in which an objective function $Q(\theta; \theta^{old})$ of the parameters θ is calculated given some previous guess θ^{old}, and an M-step, in which this function is optimized with respect to θ (see, e.g., [2] for an insightful and concise introduction). The alternating iteration of the E-step and M-step is guaranteed to converge to a local maximum of the marginal likelihood. The application of the Baum-Welch algorithm to dsHMMs, however, is not straightforward and requires some adaptations. Our sparse parametrization of the transition matrix and the emission distributions introduce additional non-linear constraints, which make the maximization of the objective function Q in the M-step a non-convex optimization problem.

4.1 The E-step

The function $Q(\theta;\theta^{old})$ for an HMM is given by

$$Q(\theta;\theta^{old}) = \sum_{\mathcal{S}=(s_1,\dots,s_T)\in(\mathbb{D}^2)^T} P(\mathcal{S} \mid \mathcal{O},\theta^{old}) \cdot \log P(\mathcal{O},\mathcal{S} \mid \theta^{old}) \tag{18}$$

First, the terms $\alpha_t(s)$, $\beta_t(s)$, $\gamma_t(s)$, $\zeta_t(r,s)$ are calculated for $s \in \mathbb{D}^2$, $t = 1,...,T-1$ as described in Section 3, using θ^{old} as parameter set. The summation over all state sequences $s \in S$ is infeasible, because $\mathcal{S}$ is prohibitively large. However, $Q(\theta;\theta^{old})$ can be represented in a way that admits its efficient computation (see [9] for details).

$$\begin{aligned} Q(\theta;\theta^{old}) &= \sum_{\mathcal{S}} P(\mathcal{S} \mid \mathcal{O},\theta^{old}) \cdot \log P(\mathcal{O},\mathcal{S} \mid \theta) \\ &= \sum_{s\in\mathbb{D}^2} \gamma_1(s)\log\tau_s + \sum_{t=2}^{T}\sum_{s\in\mathbb{D}^2}\sum_{r\in\mathbb{D}^2} \xi_{t-1}(s,r)\log b_{sr} \\ &\quad +\sum_{t=1}^{T}\sum_{s\in\mathbb{D}^2}\gamma_t(s)\log\psi_s(o_t) \end{aligned} \tag{19}$$

We introduce some auxiliary terms $\gamma_t^+(s^+)$, $\gamma_t^-(s^-)$, $\xi_t^+(r^+,s^+)$, and $\xi_t^-(r^-,s^-)$ which are strand-specific variants of $\gamma_t(s)$ respectively $\xi_t(r,s)$, $r=(r^+,r^-)$, $s=(s^+,s^-)\in\mathbb{D}^2$:

$$\begin{aligned} \gamma_t^+(s^+) &= P(s_t^+ = s^+ \mid \mathcal{O},\theta^{old}) = \sum_{s^-\in D^-} P(s_t=(s^+,s^-) \mid \mathcal{O},\theta^{old}) \\ &= \sum_{s^-\in D^-}\gamma_t(s^+,s^-) \end{aligned} \tag{20}$$

$$\gamma_t^-(i^-) = P(s_t^- = i^- \mid \mathcal{O},\theta^{old}) = \sum_{i^+\in D^+}\gamma_t(i^+,i^-) \tag{21}$$

Further,

$$\begin{aligned} \xi_t^+(i^+,j^+) &= P(s_t^+ = i^+, s_{t+1}^+ = j^+ \mid \mathcal{O},\theta^{old}) \\ &= \sum_{i^-\in D^-}\sum_{j^-\in D^-} P(s_t=(i^+,i^-), s_{t+1}=(j^+,j^-) \mid \mathcal{O},\theta^{old}) \\ &= \sum_{i^-\in D^-}\sum_{j^-\in D^-} \xi_t((i^+,i^-),(j^+,j^-)) \end{aligned} \tag{22}$$

$$\xi_t^-(i^-,j^-) = P(s_t^- = i^-, s_{t+1}^- = j^- \mid \mathcal{O},\theta^{old}) = \sum_{i^+\in D^+}\sum_{j^+\in D^+}\xi_t((i^+,i^-),(j^+,j^-)) \tag{23}$$

Insertion of (20,21,22,23) into Equation 19 leads to

$$
\begin{aligned}
Q(\theta;\theta^{old}) &= \sum_{s^+\in\mathbb{D}} \gamma_1(s^+)\log\pi_{s^+} + \sum_{s^-\in\mathbb{D}} \gamma_1(s^-)\log\pi_{s^-} \\
&\quad \sum_{t=2}^{T}\sum_{s^+\in\mathbb{D}}\sum_{r^-\in\mathbb{D}} \xi_{t-1}(s^+,r^+)\log a_{r^+s^+} + \sum_{t=2}^{T}\sum_{s^-\in\mathbb{D}}\sum_{r^-\in\mathbb{D}} \xi_{t-1}(s^-,r^-)\log a_{s^-r^-} \\
&\quad +\sum_{t=1}^{T}\sum_{s\in\mathbb{D}^2} \gamma_t(s)\log\psi_s(o_t) \\
&= \sum_{j\in\mathbb{D}} \left(\gamma_1^+(j)\cdot\log\pi_j + \gamma_T^-(j)\cdot\log\pi_j\right) \\
&\quad +\sum_{t=2}^{T}\sum_{j\in\mathbb{D}}\sum_{k\in\mathbb{D}} \left(\xi_{t-1}^+(j,k)\cdot\log a_{jk} + \xi_{t-1}^-(j,k)\cdot\log a_{kj}\right) \\
&\quad +\sum_{t=1}^{T}\sum_{s\in\mathbb{D}^2} \gamma_t(s)\log\psi_s(o_t) \qquad (24)
\end{aligned}
$$

4.2 The M-step for the transition probabilities

It is not possible to maximize Q independently for π and A as they depend on each other via the stationary state condition $\pi A = \pi$. It seems difficult to give an analytical solution for this constrained optimization problem, at least we were unsuccessful in our attempts. We therefore adopt a two-step strategy: First, we simply ignore the dependency of π and A, because the influence of π is negligible anyway once the observation sequence $\mathcal{O}$ is long enough. An analytical update is then easily to obtained by introducing Lagrange multipliers $\lambda_j(1-\sum_k a_{jk})$, $j\in\mathbb{D}$ and $\tau(1-\sum_k \pi_k)$, and setting to zero the partial derivatives of Q with respect to π and A:

$$0 = \frac{\partial}{\partial\pi_j} Q_{obs}(\theta;\theta^{old}) = \frac{\gamma_1^+(j)+\gamma_T^-(j)}{\pi_j} - \tau \qquad (25)$$

Thus, $\tau = \sum_{j\in\mathbb{D}} \tau\pi_j = \sum_{j\in\mathbb{D}}(\gamma_1^+(j)+\gamma_T^-(j)) = 2$, and

$$\pi_j = \frac{\gamma_1^+(j)+\gamma_T^-(j)}{2} \quad , \ j\in\mathbb{D} \qquad (26)$$

Similarly,

$$0 = \frac{\partial}{\partial a_{jk}} Q_{obs}(\theta;\theta^{old}) = \frac{\sum_{t=2}^{T}(\xi_{t-1}^+(j,k)+\xi_{t-1}^-(k,j))}{a_{jk}} - \lambda_j \qquad (27)$$

The last equation implies $\lambda_j = \sum_{k\in\mathbb{D}} \lambda_j a_{jk} = \sum_{k\in\mathbb{D}}\sum_{t=2}^{T}(\xi_{t-1}^+(j,k)+\xi_{t-1}^-(k,j)) = \sum_{t=2}^{T}(\gamma_{t-1}^+(j)+\gamma_t^-(j))$, and

$$a_{jk} = \frac{\sum_{t=2}^{T}(\xi_{t-1}^+(j,k)+\xi_{t-1}^-(k,j))}{\sum_{t=2}^{T}(\gamma_{t-1}^+(j)+\gamma_t^-(j))} \qquad (28)$$

Second, this solution serves as an initialization to `solnp`, an algorithm for constrained optimization problems from the R package *Rsolnp* [11, 3]. In the course of the Baum-Welch algorithm some transitions can become very unlikely. The numerical optimization gets unstable when trying to estimate these low values. Thus, we exclude transitions which are extremely rare from the maximization step and set their corresponding transition probabilities to 0. If the sum $\sum_{t=2}^{T}(\xi_{t-1}^+(i,j)+\xi_{t-1}^-(j,i))$ is smaller than 1, we set $a_{jk}=0$. This seems to be reasonable since this sum represents the number of times that we expect state i to be followed by state j on one of the two strands.

4.3 The M-step for the emission distributions

The means ν_r^s, $s\in\mathbb{D}^2$, $r\in P$ of the tuple states are, by Equation (11), defined in terms of the parameters $\mu_r^j = \begin{pmatrix}\mu_B^j\\ \mu_E^j\end{pmatrix}$, $j\in\mathbb{D}$, $r\in B\cup E$. Let $r(p)\in E\cup B$ and $j(s,p)\in\mathbb{D}$ be uniquely defined such that $\nu_p^s = \mu_{r(p)}^{j(s,p)}$. We

need to calculate the derivative of Q with respect to μ_r^j , for $r \in E \cup B$ and $j \in \mathbb{D}$.

$$
\begin{aligned}
\frac{\partial}{\partial \mu_r^j} Q(\theta;\theta^{old}) &= -\frac{1}{2} \cdot \sum_{s\in\mathbb{D}^2} \sum_{t=1}^{T} \gamma_t(s) \cdot \frac{\partial}{\partial \mu_r^j} \left[(o_t - \nu^s)^T (\Gamma^s)^{-1} (o_t - \nu^s) \right] \\
&\overset{\text{product rule}}{=} - \sum_{s\in\mathbb{D}^2} \sum_{t=1}^{T} \gamma_t(s) \cdot (o_t - \nu^s)^T (\Gamma^s)^{-1} \left[\frac{\partial}{\partial \mu_r^j} (o_t - \nu^s) \right] \\
&= \sum_{s\in\mathbb{D}^2} \sum_{t=1}^{T} \gamma_t(s) \cdot (o_t - \nu^s)^T (\Gamma^s)^{-1} \frac{\partial \nu^s}{\partial \mu_r^j} \qquad (29)
\end{aligned}
$$

Further,

$$
\frac{\partial \nu_p^s}{\partial \mu_r^j} = \frac{\partial \mu_{r(p)}^{j(s,p)}}{\partial \mu_r^j} = \delta_{r=r(p)} \cdot \delta_{j=j(s,p)} \qquad (30)
$$

Insertion of (30) into (29) leads to

$$
\begin{aligned}
\frac{\partial}{\partial \mu_r^j} Q(\theta;\theta^{old}) &= \sum_{s\in\mathbb{D}^2} \sum_{t=1}^{T} \gamma_t(s) \cdot (o_t - \nu^s)^T (\Gamma^s)^{-1} \left(\delta_{r=r(p)} \cdot \delta_{j=j(i,p)} \right)_{p\in P} \\
&= \sum_{s\in\mathbb{D}^2} \sum_{t=1}^{T} \gamma_t(s) \cdot \sum_{p_1\in P} \sum_{p_2\in P} (o_{t,p_1} - \mu_{r(p_1)}^{j(s,p_1)}) (\Gamma^s)^{-1}_{p_1 p_2} \left(\delta_{r=r(p_2)} \cdot \delta_{j=j(i,p_2)} \right) \\
&= \sum_{p_2\in P} \sum_{\substack{s\in\mathbb{D}^2 \\ j(s,p_2)=j}} \sum_{t=1}^{T} \gamma_t(s) \cdot \sum_{p_1\in P} (o_{t,p_1} - \mu_{r(p_1)}^{j(s,p_1)}) (\Gamma^s)^{-1}_{p_1 p_2} \left(\delta_{r=r(p_2)} \right) \\
&= \sum_{\substack{p_2\in P \\ r(p_2)=r}} \sum_{\substack{s\in\mathbb{D}^2 \\ j(s,p_2)=j}} \sum_{t=1}^{T} \gamma_t(s) \cdot \sum_{p_1\in P} (o_{t,p_1} - \mu_{r(p_1)}^{j(s,p_1)}) (\Gamma^s)^{-1}_{p_1 p_2} \qquad (31)
\end{aligned}
$$

Setting (31) to zero leads to

$$
\begin{aligned}
\sum_{\substack{p_2\in P \\ r(p_2)=r}} \sum_{\substack{s\in\mathbb{D}^2 \\ j(s,p_2)=j}} \sum_{t=1}^{T} \gamma_t(s) \cdot \sum_{p_1\in P} o_{t,p_1} (\Gamma^s)^{-1}_{p_1 p_2} &= \sum_{\substack{p_2\in P \\ r(p_2)=r}} \sum_{\substack{s\in\mathbb{D}^2 \\ j(s,p_2)=j}} \sum_{t=1}^{T} \gamma_t(s) \cdot \sum_{p_1\in P} (\Gamma^s)^{-1}_{p_1 p_2} \cdot \mu_{r(p_1)}^{j(s,p_1)} \\
\underbrace{\sum_{\substack{p_2\in P \\ r(p_2)=r}} \sum_{\substack{s\in\mathbb{D}^2 \\ j(s,p_2)=j}} \sum_{t=1}^{T} \gamma_t(s) \cdot o_t^T \cdot (\Gamma^s)^{-1}_{p_1 p_2}}_{b_{(j,r)}} &= \sum_{\substack{p_2\in P \\ r(p_2)=r}} \sum_{\substack{s\in\mathbb{D}^2 \\ j(s,p_2)=j}} \sum_{p_1\in P} \underbrace{\sum_{t=1}^{T} \gamma_t(s) \cdot (\Gamma^s)^{-1}_{p_1 p_2}}_{c^s_{p_1 p_2}} \cdot \mu_{r(p_1)}^{j(s,p_1)} \qquad (32)
\end{aligned}
$$

Equation (32) is linear in the parameters μ_r^j. To finally determine the means μ_r^j, $j \in \mathbb{D}$, $r \in E \cup B$ we set up a matrix equation, which can then be solved by standard matrix algebra. Let $b = (b_{(j,r)})_{(j,r)}$, $\mu = (\mu_r^j)_{(j,r)}$, $m = |\mathbb{D}| \cdot |E \cup B|$. Assuming an arbitrary (fixed) order of the index tuples (j,r), we treat b and μ as column vectors. We construct an $m \times m$ matrix H by setting

$$
H_{(j,r),(j',r')} = \sum \{ c^s_{p_1 p_2} \mid p_2 \in P, r(p_2) = r, s \in \mathbb{D}^2, j(s,p_2) = j, p_1 \in P, \mu_{r(p_1)}^{j(s,p_1)} = \mu_{r'}^{j'} \} \qquad (33)
$$

with the convention that this sum is zero when it is empty.

Check that for $j \in \mathbb{D}$, $r \in E \cup B$

$$
\begin{aligned}
& \sum_{(j',r') \in \mathbb{D} \times (E \cup B)} H_{(j,r)(j',r')} \mu_{(j',r')} \\
= & \sum_{j' \in \mathbb{D}} \sum_{r' \in E \cup B} \sum \{ c^s_{p_1 p_2} \mid p_2 \in P, r(p_2) = r, s \in \mathbb{D}^2, j(s,p_2) = j, p_1 \in P, \mu^{j(s,p_1)}_{r(p_1)} = \mu^{j'}_{r'} \} \mu^{j'}_{r'} \\
\overset{(*)}{=} & \sum \{ c^s_{p_1 p_2} \mu^{j(s,p_1)}_{r(p_1)} \mid p_2 \in P, r(p_2) = r, s \in \mathbb{D}^2, j(s,p_2) = j, p_1 \in P \} \\
= & \sum_{\substack{p_2 \in P \\ r(p_2) = r}} \sum_{\substack{s \in \mathbb{D}^2 \\ j(s,p_2) = j}} \sum_{p_1 \in P} c^s_{p_1 p_2} \mu^{j(s,p_1)}_{r(p_1)} \\
\overset{(46)}{=} & b_{(j,r)}
\end{aligned} \tag{34}
$$

(*) note that $\mu^{j(s,p_1)}_{r(p_1)} = \mu^{j'}_{r'}$ exactly for one choice of j' and r'.

The conditions in (32) can be then summarized (and solved) conveniently as one matrix equation,

$$b = H\mu \tag{35}$$

For the update of the means we need to solve the linear equation in (35). To do so we employ the function `solve` of the R base package [8]. However, the linear system has no unique solution if the coefficient matrix H is singular. This can happen in the following situation: Let $j \in \mathbb{D}$, $r \in B$ be fixed. Due to (33) all entries in row (j,r) of matrix H are zero if there is no $s \in \mathbb{D}^2$ and no $p_2 \in P$ such that $j(s,p_2) = j$ and $r(p_2) = r$. This is the case when μ^j_r does not occur in any $\nu^s_{p_2}$, i.e. when state j does not dominate the occupancy of observation r in combination with any state. Consequently, also all entries in column (j,r) of matrix H are zero as for the same reason there is no $p_1 \in P$ fulfilling $\mu^{j(s,p_1)}_{r(p_1)} = \mu^j_r$. Usually, this scenario cannot happen as μ^j_r occurs at least in the means of state $s = (j,j)$. However, since we successively exclude rare tuple states from the learning process in order to improve runtime (see section 6), state $s = (j,j)$ does not need to be part of the model until the end of the training process. Hence, it is possible that some rows of H (and the corresponding columns) are zero. In this case H is singular. In order to avoid singularity of H, we exclude all parameters μ^j_r that do not occur in any tuple means from the maximization step. I.e., we remove the corresponding rows and columns from H and the corresponding entries from $\mu_{(j,r)}$ and $b_{(j,r)}$. As a consequence, the remaining $\mu_{(j,r)}$ can be updated by employing the `solve` function. The parameters $\mu_{(j,r)}$ which we do not update are irrelevant to the model since they do not influence the dsHMM likelihood function.

Treatment of the covariance matrices

For the estimation of the covariance matrices we cannot give an analytical solution in the M-step. In the current version the covariance matrices are not updated at all. Instead, we conservatively fix the covariance matrix Γ^s of each state to the empirical covariance of the whole data,

$$\Gamma = \sum_{t=1}^{T} (o_t - \nu)^T (o_t - \nu)$$

where $\nu = \frac{1}{T} \sum_{t=1}^{T} o_t$ is the global mean of the data.

Another approach would be to adapt the learning of covariance matrices in standard HMMs, ignoring dependencies introduced by the dsHMM, $\Gamma^s = \sum_{t=1}^{T} \gamma_t(s)(o_t - \nu^s)^T (o_t - \nu^s) / \sum_{t=1}^{T} \gamma_t(s)$. However, it is known that the unregularized update of the covariance matrices often leads to overfitting and to 'singularities', i.e., the emission distribution of a state converges to a point mass during the EM algorithm. A feasible future improvement could the learning of empirical covariance matrices of transcribed states within regions that show transcriptional activity only on one strand. We experienced that the optimization of the covariance matrices, if done cautiously, has only a minor impact on the results, and therefore is not further pursued here.

Multiple observation sequences

All equations we have deduced in the previous sections handle a single observation sequence. When applying dsHMMs to genomics data, however, we encounter multiple observation sequences, $\mathcal{O} = \{\mathcal{O}^1, \mathcal{O}^2, ..., \mathcal{O}^N\}$. As

before the aim is to maximize $P(\mathcal{O} \mid \theta) = \prod_{n=1}^{N} P(\mathcal{O}^n \mid \theta)$ with respect to θ, assuming independence of observation sequences. This can be done in a straightforward manner (see, e.g., Rabiner [9]). The target function Q is modified into a sum of N individual Q functions as given in (24), one for each observation sequence $\mathcal{O}^n$, $n = 1, ..., N$.

5 The Viterbi algorithm for dsHMMs

The Viterbi algorithm calculates the most likely hidden state sequence that emitted a given sequence of observations $\mathcal{O} = (o_1, ..., o_T)$. The Viterbi algorithm for dsHMMs is identical to the standard Viterbi algorithm. We reproduce it here for the sake of completeness. For $s \in \mathbb{D}^2$, $t = 1, ..., T$, we define

$$\delta_t(s) = \max_{s_1, s_2, ..., s_{t-1} \in \mathbb{D}^2} P(s_1, s_2, ..., s_{t-1}, s_t = s, o_1, ..., o_t \mid \theta) \tag{36}$$

I.e., $\delta_t(s)$ denotes the probability of the most likely hidden state sequence ending at time t in state s while observing $o_1, ..., o_t$. $\delta_t(s)$ can be calculated recursively by

$$\begin{aligned} \delta_1(s) &= \pi_s \psi_s(o_1) \quad , \ s \in \mathbb{D}^2 \\ \delta_t(s) &= \left[\max_{r \in \mathbb{D}^2} \delta_{t-1}(r) \cdot b_{rs} \right] \psi_s(o_t) \quad , \ s \in \mathbb{D}^2, \ t = 2, ..., T \end{aligned} \tag{37}$$

In order to obtain not only the probabilities but also the hidden state sequence itself, we need to save the predecessor state which maximized the term $\delta_t(s)$ for each t and s. To do so, we introduce the auxiliary variable $h_t(s)$:

$$\begin{aligned} h_1(s) &= 0 \quad , \ s \in \mathbb{D}^2 \\ h_t(s) &= \arg\max_{r \in \mathbb{D}^2} \delta_{t-1}(r) b_{rs} \quad , \ s \in \mathbb{D}^2, \ t = 2, ..., T \end{aligned} \tag{38}$$

The Viterbi path is then obtained by backtracking,

$$\begin{aligned} s_T &= \arg\max_{s \in \mathbb{D}^2} \delta_T(s) \\ s_t &= h_{t+1}(s_{t+1}) \quad , \ t = T-1, ..., 1 \end{aligned} \tag{39}$$

6 Limitations and alternatives to the dsHMM model

Learning and interpretation of emission distributions

The crucial choice in the construction of the emission distribution ψ_s is the rule for the means ν^s, Equation 11. Obvious, alternative choices for Equation (11) would be

$$\nu_p^s = \begin{cases} \mu_p^{s^+} + \mu_p^{s^-} & \text{if } p \in B \\ \mu_p^{s^+} & \text{if } p \in E^+ \\ \mu_p^{s^-} & \text{if } p \in E^- \end{cases} \quad , \text{ or } \quad \nu_p^s = \begin{cases} f(\mu_p^{s^+}, \mu_p^{s^-}) & \text{if } p \in B \\ \mu_p^{s^+} & \text{if } p \in E^+ \\ \mu_p^{s^-} & \text{if } p \in E^- \end{cases} \tag{40}$$

where $f(x, y) = \ln(\exp(x) + \exp(y))$. Both definitions suffer from the fact that the data we use as input is not anchored on an absolute scale (it is log transformed ChIP-on-chip data and therefore can be interpreted on a difference scale). An additive rescaling of our data therefore should not change our results. For this reason, both definitions are meaningless in our case. These definitions might become useful in the context of sequencing data, where absolute count data might be transformed into absolute occupancy values after careful normalization. Still, the M-step can not be given in closed form in case of the function f, which might make the transition parameter update prohibitively slow for genomics data.

Be aware that the construction of the means ν^s has implications on the interpretation of the dsHMM states in terms of the single-stranded parameters μ^j. By choosing the max function for strand-unspecific observations, we implicitly assume that the stronger of the two forward respectively reverse strand-associated processes dominates

the occupancy profile. While this is reasonable for transcription factors, this assumption might be questionable in general. E.g., promoters of genes are known to be depleted of nucleosomes. In this case a tuple state $s = (s^+, s^-)$ involves a promoter state s^+ or s^-, nucleosome eviction is the dominant process, and the minimum of the two means $\mu_p^{s^+}$, $\mu_p^{s^-}$ should be chosen as the value for ν_p^s. The current dsHMM model still correctly infers a low nucleosome occupancy for promoter states, because the (residual) nucleosome occupancy at promoters will be attributed the non-promoter state in the tuple s.

Speed and memory limitations

In each iteration of the Baum-Welch algorithm we determine the auxiliary terms in Equations (16),(17), and (20)-(23) in order to execute the maximization step. Their values are derived from the forward and backward probabilities $\alpha_t(s)$ and $\beta_t(s)$ (Equations 13 and 14). Their calculation constitutes the speed limiting step, because the time complexity scales with the number of tuple states, $|\mathbb{D}|^2$, while it scales linearly with observation length T. This problem can be alleviated by reducing the number of updated tuple states during the execution of the Baum-Welch algorithm. It is anticipated that some tuple states $s = (s^+, s^-)$ essentially never occur and hence can be removed from the set of possible states. If the expected frequency of state s, given by $\frac{1}{T}\sum_{t=1}^{T}\gamma_t(s)$, is smaller than 1, it is considered insignificant and is excluded from the model. In all following iterations the auxiliary terms of such a tuple state are set to 0. In this study where we trained a dsHMM with 20 single-strand states, i.e. 400 tuple states, on yeast data, we could exclude 237 out of the 400 transcription states during the Baum-Welch algorithm.
Values of $b_s(o_t)$, $\alpha_t(s)$ and $\beta_t(s)$ have to be saved separately for all values of $s \in \mathbb{D}^2$, $t = 1, ..., T$. The dsHMM memory scales with $T \cdot |\mathbb{D}|^2$, as opposed to the corresponding standard HMM, scaling with $T \cdot |\mathbb{D}|$. Since the state set $\mathbb{D}$ is typically small (<50), This is not a tremendous problem. For large genomes, we split our calculations into different chromosomes, and we eventually split chromosomes further, to obtain shorter observation sequences $\mathcal{O}^1, ..., \mathcal{O}^N$. Calculations for $\mathcal{O}^k$, $k = 1, ..., N$ can then be done sequentially, using the same memory. So far, memory has never been the limiting factor when learning the parameters of the dsHMM.

References

[1] Christopher M Bishop. *Pattern recognition and machine learning.* Springer, 2006.

[2] Frank Dellaert. The expectation maximization algorithm. Technical report GIT-GVU-02-20, College of Computing, Georgia Institute of Technology, Feb 2002.

[3] Alexios Ghalanos and Stefan Theussl. *Rsolnp: General Non-linear Optimization Using Augmented Lagrange Multiplier Method*, 2012. R package version 1.14.

[4] William Lee, Desiree Tillo, Nicolas Bray, Randall H Morse, Ronald W Davis, Timothy R Hughes, and Corey Nislow. A high-resolution atlas of nucleosome occupancy in yeast. *Nat. Genet.*, 39(10):1235–1244, Oct 2007.

[5] Andreas Mayer, Martin Heidemann, Michael Lidschreiber, Amelie Schreieck, Mai Sun, Corinna Hintermair, Elisabeth Kremmer, Dirk Eick, and Patrick Cramer. CTD tyrosine phosphorylation impairs termination factor recruitment to RNA polymerase II. *Science*, 336(6089):1723–1725, Jun 2012.

[6] Andreas Mayer, Michael Lidschreiber, Matthias Siebert, Kirstin Leike, Johannes Söding, and Patrick Cramer. Uniform transitions of the general RNA polymerase II transcription complex. *Nat. Struct. Mol. Biol.*, 17(10):1272–1278, Oct 2010.

[7] Dimitar B Nikolov and Stephen K Burley. RNA polymerase II transcription initiation: a structural view. *Proc. Natl. Acad. Sci.*, 94(1):15–22, Jan 1997.

[8] R Core Team. *R: A Language and Environment for Statistical Computing.* R Foundation for Statistical Computing, Vienna, Austria, 2013.

[9] Lawrence R Rabiner. A tutorial on hidden Markov models and selected applications in speech recognition. *Proceedings of the IEEE*, 77(2):257–286, Feb 1989.

[10] Thomas Verma and Judea Pearl. Equivalence and synthesis of causal models. In *Uncertainty in Artificial Intelligence: Proceedings of the Sixth Conference*, pages 220–227, 1990.

[11] Yinyu Ye. *Interior Algorithms for Linear, Quadratic, and Linearly Constrained Non-Linear Programming.* PhD thesis, Department of ESS, Stanford University, 1987.

[12] B. Zacher, M. Lidschreiber, P. Cramer, J. Gagneur, and A. Tresch. Annotation of genomics data using bidirectional hidden Markov models unveils variations in Pol II transcription cycle. *Mol. Syst. Biol.*, 10:768, 2014.

Part II

Supplemental Tables

State	Interpretation
1	peaks around TSS and pA site and represents both an initiation as well as a termination state as confirmed by the high occupancy of Pcf11 (0.65) a known termination factor and CTD phosphorylation at serines 2,5, and 7 and at the same time medium Pol2 occupancy (0.46). Being an initiation and termination state simultaneously, this state occurs in regions of the genome with a rapid successions of genes, where the transition between termination of one gene and initiation of the next is seamless.
2	is preferentially annotated within late elongation. Transcriptional activity and nucleosome occupancy are still high, typical for late elongation. Binding intensities of elongation factors (0.26-0.52) have higher means than initiation and termination factors (0.09-0.26), indicating ongoing productive elongation.
3	another state of late elongation, with high expression values and nucleosome occupancy. S2P, Y1P and Spn1, characteristic of late elongation, show the highest factor binding means (0.59-0.72). Medium occupancy of Spt5 (0.47) and Rpb3 (0.46) is consistent with productive elongation.
4	shows the highest occupancy levels of all states across all binding factors, which together with high levels of expression suggest that this state is not restricted to a single phase of the transcription cycle. It is rarely annotated, usually on genes encoding ribosomal proteins
5	is a typical termination state, peaking at the pA site. S2P (0.80) and Y1P (0.66) are enriched in comparison to S5P (0.38) and S7P (0.43), indicating a late phase of transcription.
6	is a late elongation state. High occupancy of Rpb3 (0.66) along with high expression values and high occupancy of elongation factors Spt5 and Spn1) are consistent with the phase of late elongation. Means for the termination factors Pcf11 and Nrd1 are in the lower medium range, suggesting that recruitment of termination factors has not started yet.
7	is an early elongation state. Mean occupancy of elongation factors Spt5, Bur1 and Spn1 range from 0.31 to 0.36. Means of S5P and S7P are higher than means of S2P and Y1P, indicating an early phase of transcription.
8	is an early elongation state. It is defined by medium to high mean occupancies of Rbp3 and of the elongation factors Spt5, Bur1 and Spn1. Mean Nucleosome occupancy is high (0.7).
9	is annotated on the antisense strand on regions where the sense strand is annotated by states coding for mid or late elongation. It was likely generated during the learning process to best fit the transcribed states of the opposite strand.
10	is a state of transcription initiation, peaking after the TSS. Nucleosomes, Rpb3, S5P, S7P, and the elongation factors Spt5, Bur1 and Spn1 all show high occupancy (0.62-0.83). The initiation factor TFIIB shows high occupancy means, suggesting that the state codes for situation where the elongation factors have already been recruited, but initiation has not yet finished.

Table 1: Description of states 1-10 of the learned dsHMM. We refer to protein occupancy and expression as low, medium or high, if the respective mean value is < 0.4, ≥ 0.4 and ≤ 0.6 or > 0.6 (see Figure 4, Main Text). The abbreviation CUT stands for 'cryptic unstable transcripts'.

State	Interpretation
11	represents the phase of mid elongation. Transcription is in the medium range, whereas binding intensities for all factors except nucleosomes are in the low range. S5P and S7P show lower occupancies than S2P and Y1P indicating that transcription has shifted from early to productive elongation.
12	is an intergenic state mostly assigned to transcriptionally inactive regions. It s defined by its low binding intensities for all factors (-0.07 to 0.24), except nucleosomes (0.77).
13	is a termination state peaking at the pA site. Both termination factors, Pcf11 (0.76) and Nrd1 (0.74), are increased compared to states of early, mid and late elongation.
14	is a state of mid elongation as well. Binding of the elongation factors Spt5, Bur1 and Spn1 (0.43-0.56) exceeds binding of initiation and termination factors (0.25-0.40). High nucleosome occupancy and medium Rpb3 occupancy.
15	is an transcription inititation state exhibiting a sharp peak at the TSS in the state frequency profile. Mean TFIIB occupancy is high (0.68). Rpb3 and the phosphorylated forms S5P and S7P of Pol II show medium occupancy. Low occupancies of S2P and Y1P suggest that productive transcription has not started yet.
16	is an early termination state. Occupancies of all factors, except the termination factors Nrd1 and Pcf11, are low. The state seems to be characterized by the binding of Nrd1, which shows by far the highest binding intensity with a mean of 0.51. This state is used by the model to annoted CUTs.
17	is a pre-initiation state, occurring in the promoter region of genes. This state exhibits nucleosome depletion (-0.11) and high occupancy of TFIIB (0.84). Low mean occupancies of the elongation factors, the termination factors, S2P and Y1P.
18	represents an intergenic state and all forms of Pol II show signals close to 0. The state frequency profile of the antisense strand indicates that this state is annotated on genomic regions whether there are transcribed elements on the opposite strand not. It is the most frequently annotated state (with a stationary distribution of 0.16).
19	is an intergenic state.
20	is a mixture between termination and initiation state with a stronger tendency towards termination. It shows increased binding of TFIIB (0.55) in combination with nucleosome depletion (0.16) and high termination factor binding.

Table 1 (ctd.): Description of states 11-20 of the learned dsHMM. We refer to protein occupancy and expression as low, medium or high, if the respective mean value is < 0.4, ≥ 0.4 and ≤ 0.6 or > 0.6 (see Figure 4, Main Text). The abbreviation CUT stands for 'cryptic unstable transcripts'.

Rpb3 is a subunit of Pol II and therefore required for the transcription of protein coding genes, CUTs, SUTs, small nuclear RNA and small nucleolar RNA. [6]

S5P denotes a form of Pol II that is phosphorylated at the Ser2 residues of the CTD. It is involved in the recruitment of the capping enzyme and Nrd1. The occupancy of S5P decreases over the transcribed region. [5]

S7P terms a Ser7 phosphorylated form of Pol II. Similar to S5P, it shows a decreasing occupancy over the transcribed region. [6]

Y1P is another phosphorylated form of Pol II. It shows phosphorylation at residues Tyr1 of the CTD. Y1P impairs the recruitment of termination factors, which is consistent with its occupancy profile over the gene. Binding of Y1P increases over the transcribed region and shows a decline 180 nucleotides upstream of the pA site. [5]

S2P denotes a form of Pol II phosphorylated at the Ser2 residues of the CTD. It stabilizes the elongation complex. Occupancy increases over the transcribed region. [6]

TFIIB is one of the general transcription factors required for initiation. It plays a role in the start site selection of Pol II. [7]

Spt5, Bur1, Spn1 are all elongation factors. Spt5 is a group 1 elongation factor, Bur1 a group 3 elongation factor and Spn1 is a group 2 elongation factor. Groups were distinguished according to their occupancy profiles as described in the introduction. [6]

Nrd1 is a termination factor. The occupancy profile over genes correlates with the occupancy profile of S5P indicating Nrd1 binding to the Ser5-phosphorylated CTD. This corresponds to a function of Nrd1 in early transcription termination. [5]

Pcf11 is a 3' processing and termination factor. The occupancy of Pcf11 increases within the transcribed region and peaks downstream of the pA site. [6]

Nucleosome occupancy shows a depletion at promoter regions in order to enable binding of Pol II. [4]

Table 2: Description of the factors used in training the model

Part III
Supplemetal Figures

A bdHMM was learned on 20 states, the same number of states as the strand-specific states of the dsHMM. The bdHMM was initialized with the same parameters as the dsHMM. Among the 20 states, 5 states initialized from data of untranscribed regions were declared undirected, whereas the 15 states learned on transcribed regions were declared directed (for a thorough description of the bdHMM, see [12]). For a set of 4362 representative genes that was also used for the construction of Figure 4 from the main text, we compared the bdHMM state annotation to the dsHMM state annotation of the sense strand (2A) and the antisense strand (2B) of the respective gene. Each matrix entry m_{jk} encodes the relative frequency of the occurrence of dsHMM state k (column k), given that it was annotated as state j by the bdHMM (row j). In other words, the two matrices are frequency matrices in which each row has been normalized to sum one. To better illustrate the dsHMM states and their putative function, we added the transition graph from Main Figure 4 below each matrix. Colored nodes correspond to "transcription" states thought to be involved in mRNA transcription, grey nodes correspond to "intergenic" states. After appropriate renumbering of the bdHMM states, the matrix in 2A is essentially a diagonal matrix. Thus, the directed bdHMM states correspond very well to a unique sense strand dsHMM state. Moreover, these dsHMM states are transcription states. The undirected bdHMM states show a moderate agreement with the remaining intergenic dsHMM states on the sense strand. This indicates that the bdHMM annotation agrees well with the sense strand dsHMM annotation in genomic positions with only one ongoing directed process. This is supported by the observation that in the matrix in 2B, directed bdHMM states mainly align to intergenic (transcriptionally silent) dsHMM states on the antisense strand. Further, the agreement of the undirected bdHMM states with their corresponding intergenic dsHMM states on the antisense strand is as good as the agreement on the sense strand, indicating that these regions are in fact transcriptionally silent. However, the undirected states 17, 18, and 19 are essentially the only states which are occasionally associated with transcribed antisense dsHMM states. This indicates that transcription in one direction generally impedes simultaneous transcription in the opposite direction.

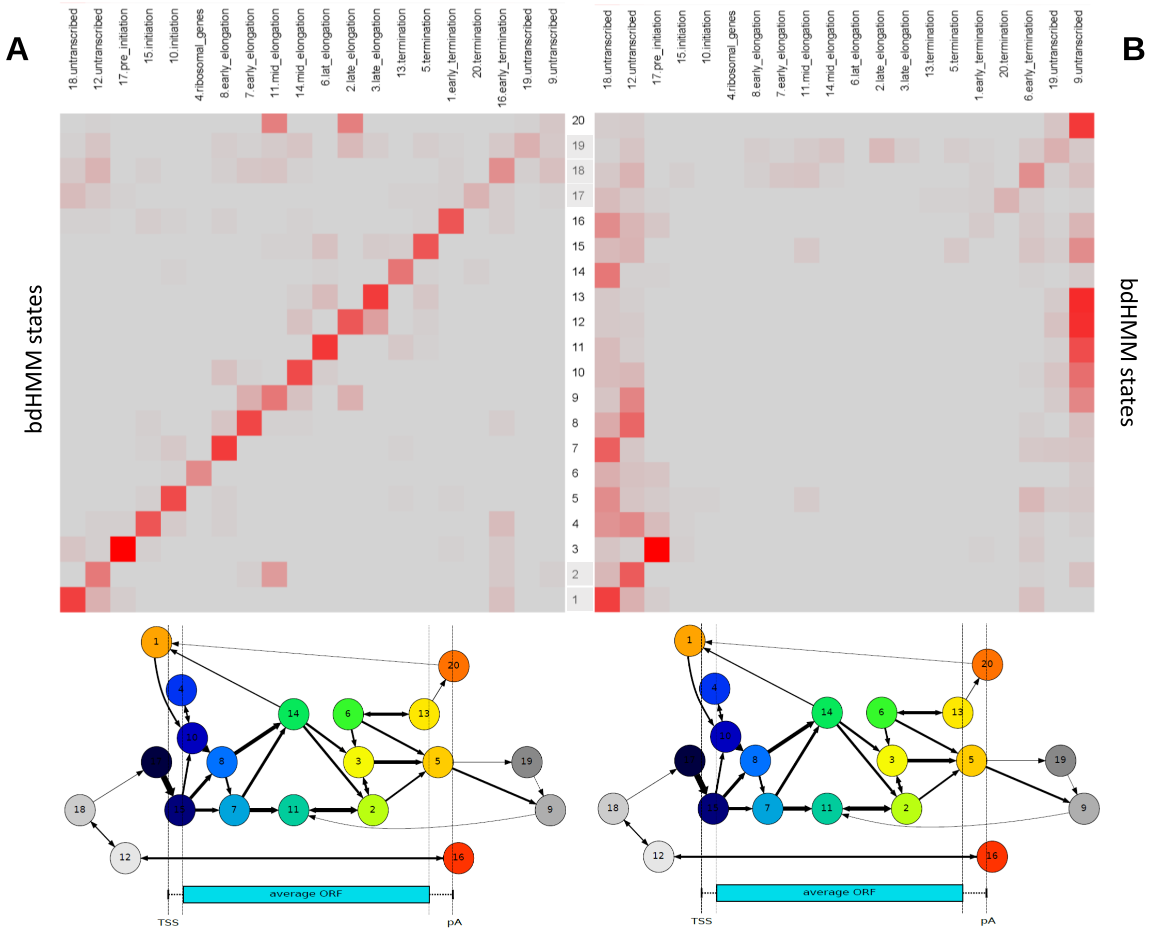

Figure 2: Comparison of the genome annotation obtained by the dsHMM and the bdHMM. The bdHMM was initialized with the same parameters as the dsHMM. Among the 20 states, 5 states initialized from data of untranscribed regions were declared undirected (grey boxed numbers on the y-axis), whereas the 15 states learned on transcribed regions were declared directed. For a set of 4362 representative genes that was also used for the construction of Figure 4 from the main text, we compared the bdHMM state annotation to the dsHMM state annotation of the sense strand (A) and the antisense strand (B) of the respective gene. The ds HMM transition graph (Figure 4, mein text) is shown below the matrix. Colored nodes correspond to “transcription” states thought to be involved in mRNA transcription, grey nodes correspond to “intergenic” states.
